# Adversarial Robustness of Deep Reinforcement Learning Based Dynamic Recommender Systems

**DOI:** 10.3389/fdata.2022.822783

**Published:** 2022-05-03

**Authors:** Siyu Wang, Yuanjiang Cao, Xiaocong Chen, Lina Yao, Xianzhi Wang, Quan Z. Sheng

**Affiliations:** ^1^School of Computer Science and Engineering, University of New South Wales, Sydney, NSW, Australia; ^2^School of Computer Science, University of Technology Sydney, Sydney, NSW, Australia; ^3^Department of Computing, Macquarie University, Sydney, NSW, Australia

**Keywords:** recommender systems (RS), deep reinforcement learning (deep RL), adversarial attack, robustness, deep learning—artificial neural network (DL-ANN)

## Abstract

Adversarial attacks, e.g., adversarial perturbations of the input and adversarial samples, pose significant challenges to machine learning and deep learning techniques, including interactive recommendation systems. The latent embedding space of those techniques makes adversarial attacks challenging to detect at an early stage. Recent advance in causality shows that counterfactual can also be considered one of the ways to generate the adversarial samples drawn from different distribution as the training samples. We propose to explore adversarial examples and attack agnostic detection on reinforcement learning (RL)-based interactive recommendation systems. We first craft different types of adversarial examples by adding perturbations to the input and intervening on the casual factors. Then, we augment recommendation systems by detecting potential attacks with a deep learning-based classifier based on the crafted data. Finally, we study the attack strength and frequency of adversarial examples and evaluate our model on standard datasets with multiple crafting methods. Our extensive experiments show that most adversarial attacks are effective, and both attack strength and attack frequency impact the attack performance. The strategically-timed attack achieves comparative attack performance with only 1/3 to 1/2 attack frequency. Besides, our white-box detector trained with one crafting method has the generalization ability over several other crafting methods.

## 1. Introduction

Recommendation systems are an effective means of alleviating information overload for Internet users. They generally filter out those less irrelevant ones from massive items of choice to improve user experience in multiple scenarios. Traditional recommendation systems extract features about user preferences, items, and users' past interactions with items to conduct content-based, collaborative, or hybrid recommendations (G.Adomavicius and A.Tuzhilin, 2005; Zhang et al., 2019). These models have not considered the changes in user preferences over time. In this regard, interactive recommendation systems emerge to capture personalized user preference dynamics. Generally, interactive recommendation systems cater to users' dynamic and personalized requirements by improving the rigid strategy of conversational recommendation systems (Thompson et al., 2004; Mahmood and Ricci, 2007; Taghipour and Kardan, 2008). In recent years, they have been attracting increasing attention and employed in leading companies (e.g., Amazon, Netflix, and YouTube) for personalized recommendations.

Interactive recommendation systems can be considered a decision-making process where the system chooses an optimal action in each discrete step to maximize the user response evaluation. Common practices to model the interactions between recommendation systems and users include Multi-Armed Bandit (MAB) or Reinforcement Learning (RL). The former views the action choice as a repeated single process while the latter considers immediate and future rewards to better model long-term user preference behaviors. In RL-based recommender systems, a Markov Decision Process (MDP) agent estimates the value function by both action and state rather than merely by action as done by MAB.

However, small disturbances in the input data may fool the above practices (Szegedy et al., 2013; Goodfellow et al., 2015). Small imperceptible noises, such as adversarial examples, may increase prediction error or reduce reward in supervised and RL tasks—the input noise can be transferred to attack different parameters even different models, including recurrent network and RL (Huang et al., 2017; Gao et al., 2018). Besides, the vector representations of entity/relation embedding of the input of RL-based recommendation models make it challenging for humans to tell the true value or dig out the real issues in the models.

Recently, Browne and Swift (2020) point out that counterfactual reasoning can be used to generate adversarial samples. From the perspective of causal inference, one way to leverage counterfactual reasoning is by intervening on some causes in the data generation process to generate adversarial samples. Since both perturbations and counterfactual reasoning target the state space by introducing noise, attackers can easily leverage such characteristics of embedding vectors to disrupt recommendation systems silently. Therefore, it is crucial to study attack and defense methods for RL-based recommendation systems.

This study aims to develop a general detection model to detect attacks and increase the defense ability and robustness, which provides a practical strategy to overcome the dynamic “arm-race” of attack and defense in the long run. The problem is nontrivial due to three reasons. First, online attacks are inherently difficult to track or predict. Second, man-in-middle methods can attack the interactions between recommendation systems and users in web applications, giving opportunities for malicious people to disrupt recommendation systems in either a white-box or a black-box way. Third, the vast number of actions in RL-based recommendation systems poses a barrier to detecting user feedback since the exhaustively numerous items and user embedding vectors are not feasible to find the abnormal inputs. We propose an attack-agnostic detection model against adversarial examples for RL-based recommendation systems to overcome the above challenges. To the best of our knowledge, this is the study that focuses on the adversarial detection of RL-based Recommendation Systems. We make the following contributions:

- We systematically investigate different types of adversarial attacks and detection approaches focusing on RL-based recommendation systems and demonstrate the effectiveness of the designed adversarial examples and strategically-timed attacks.
- We propose an encoder-classification detection model for attack-agnostic detection. The encoder captures the temporal relationship among sequence actions in RL. We further use an attention-based classifier to highlight the critical time steps out of a large interactive space.
- We empirically show that even small perturbations or counterfactual states can significantly reduce most attack methods' performance. Our statistical validation shows that multiple attack methods generate similar actions of the attacked system, providing insights for improving the efficacy of the detection performance.

## 2. Related Study

**RL-based interactive recommendation**. RL is a popular approach to the interactive recommendation. Traditional research applies Q-learning (Taghipour et al., 2007; Taghipour and Kardan, 2008) and MDP (Mahmood and Ricci, 2009) to web recommendation and conversational recommendation problems. Mahmood and Ricci (2007) first introduce reinforcement learning into an interactive recommendation by modifying MDP. Since then, deep learning has inspired more interest in the interactive recommendation. For example, Christakopoulou et al. (2018) employ reinforcement learning to improve feedback quality in interactive recommendation; Chen et al. (2019) adopt policy gradient to improve the scalability of interactive recommendation.

**Adversarial attacks**. We explore the test-time white-box attack for the RL-based recommender system. This branch of study starts from Szegedy et al. (2013), which first find that hardly perceptible perturbation can cause erroneous outputs of a convolutional neural network on image classification tasks. Goodfellow et al. (2015) exploit this topic further and incorporate the Fast Gradient Sign Method to attack neural networks; (Papernot et al., 2016) proposes Jacobian-based Saliency Map Attack (JSMA) algorithm to greedily select attack pixels by Jacobian matrix. Another view is to model the attack as an iterated optimization process, like the Deepfool model (Moosavi-Dezfooli et al., 2016) and PGD (Kurakin et al., 2016). Croce and Hein (2020) use the approximated hyperplane as Deepfool and guarantee the perturbed data point is closed to the hyperplane. Yu et al. (2021) exploits the feature level attack to achieve decent results. Chen and Gu (2020) factorize pixels into two variables and create a model that can control the sparsity of the attack. Specifically, Lin et al. (2017) design strategically-timed attacks and craft deceptive images to induce the agent to make the desired actions. Browne and Swift (2020) argue that counterfactual explanations produce adversarial examples in DL research, which modify the input to cause misclassification of the network. Huang et al. (2017) explore the adversarial attack deep Q network in video game playing and conclude that retraining with adversarial examples can make the network more robust. Another thread of research applies adversarial attacks into environments for robust adversarial training. They either regard the attack as a destabilizing force to break the balance of agents in 3D scenarios (Pinto et al., 2017) or develop adversarial agents in multi-agent tasks during RL (Gleave et al., 2019). Generally, creating adversarial examples helps reduce the reward of on DQN and DDPG (Pattanaik et al., 2018), and a detection method can help better explore the potential of adversarial examples and make agents more robust in a dynamic process.

**Adversarial example detection**. Many adversarial detection methods are vulnerable to the loss functions targeted to fool them (Carlini and Wagner, 2017). Bendale et al. Bendale and Boult (2016) present OpenMax to estimate the probability of data input from unknown classes explicitly. Since then, researchers have proposed a statistical approach (Hendrycks and Gimpel, 2016), binary classification approach (Metzen et al., 2017), outlier detection approach (Grosse et al., 2017), and history queries-based approach (Chen et al., 2020) to detect adversarial examples. Our study differs from Chen et al. (2020) in exploiting the nature of RL besides query-based white-box attacks. Detection models classify the benign samples and adversarial samples by discrepancy, which is verified in many areas (Cohen et al., 2020; Esmaeilpour et al., 2020; Vacanti and Van Looveren, 2020; Massoli et al., 2021), in this article, we exert the discrepancy in action space to detect adversarial samples, which turns out to be effective for multiple attack methods.

## 3. Methodology

**Figure 1:**
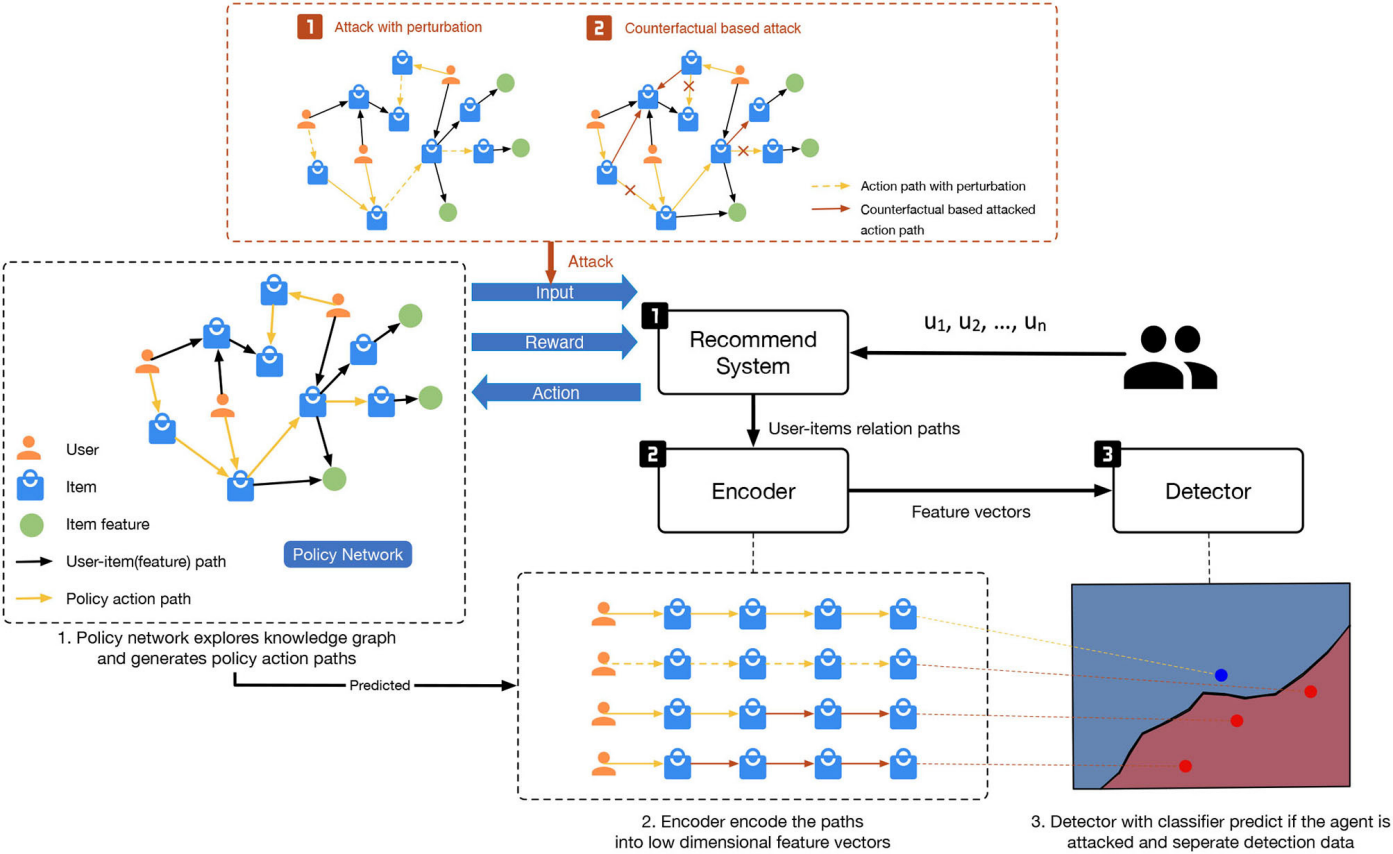
Our proposed Adversarial Attack and Detection Approach for RL-based Recommender Systems.

This section introduces the components of an RL-based recommendation system, attack techniques that generate adversarial examples, and our scheme to detect white-box adversarial attacks. The overall structure can be found in Figure 1.

### 3.1. RL-Based Interactive Recommendation

Interactive recommendation systems suggest items to users and receive feedback. Given a user *u*_*j*_ ∈ *U* = {*u*_0_, *u*_1_, *u*_2_, …, *u*_*n*_}, a set of items *I* = {*i*_0_, *i*_1_, *i*_2_, …, *i*_*n*_}, and the user feedback history *i*_*k*_1__, *i*_*k*_2__, …, *i*_*k*_*t*−1__, the recommendation system suggests a new item *i*_*k*_*t*__. This problem can be regarded as an MDP, which comprises the following:

- State (*s*_*t*_): a historical interaction between the user and the recommendation system computed by an embedding or encoder module.
- Action (*a*_*t*_): an item or a set of items recommended by the RL agent.
- Reward (*r*_*t*_): a variable related to user's feedback to guide the reinforcement model toward true user preference.
- Policy (π(*a*_*t*_|*s*_*t*_)): a probabilistic model consisting of an estimation and action generation parts. The training process aims to obtain an optimal policy for the recommendation.
- Value function (*Q*(*s*_*t*_, *a*_*t*_)): the agent's prediction of the reward of the current recommended item *a*_*t*_.

The reinforcement agent could be an Actor–Critic Algorithm that consists of a critic network and an actor network (Xian et al., 2019). The attack model may generate adversarial examples using either the critic network (Huang et al., 2017) or the actor network (Pattanaik et al., 2018).

### 3.2. Attack Model

**FGSM-based attack**. We define an adversarial example as a little perturbation δ added onto the benign examples *x* to reduce the cumulative reward of an RL system. Suppose *x* is a sequence of feature vectors piped into RL model π(*s*_*t*_); *x* can be a composition of embedding vectors of users, relations, and items (Xian et al., 2019), or a feature vector encoded user and item information (Chen et al., 2019). Unlike perturbations on images or texts, δ can be large in interactive recommendation systems due to the enormous manual work to check the embedding vectors or feature vectors of massive users and items. We define an adversarial example as follows:


(1) $$\begin{array}{l} \begin{array}{c} \underset{\delta }{\min}R_{T}=\overset{T}{\underset{t=1}{\sum }}r_{t},\\ r_{t}=Q\left(s_{t}+\delta ,\;a_{t}\right),\\ a_{t}=\pi^{*}\left(a_{t}|s_{t}+\delta \right),\\ \text{subject to}\;S\left(s_{t},s_{t}+\delta \right)\leq l,1 \end{array} \end{array}$$

where *R*_*T*_ is the total reward of the recommendation agent, *T* is the length of a time step, π^*^ is the optimal policy learned by the training process, *S* (< *l*) is a similarity metric that measures the distance between benign and adversarial examples. *S* is commonly defined as *l*_*p*_ bounded perturbation, or |δ|_*p*_ (Carlini et al., 2019). The computation of δ determines the method of attack. We aim to build a model with the generalization ability to detect examples from unknown adversarial distributions. Thus, we adopt three attack methods to validate the detection model performance: FGSM (Goodfellow et al., 2015) and its variant (Huang et al., 2017), JSMA (Papernot et al., 2016), and Deepfool (Moosavi-Dezfooli et al., 2016). FGSM can be presented as follows:


(2) $$\begin{array}{l} \delta_{inf}=\epsilon \;sign\left(\nabla_{s_{t}}J\left(Q_{t},Q_{t-1}+r_{t}\right)\right), \end{array}$$

where *J* is the loss function, *Q*_*t*_ is the critic function *Q*(*s*_*t*_, *a*_*t*_). Optimizing *J* will lead to the critic value *Q* satisfying the Bellman equation. The FGSM method uses the gradient of the loss function, which can be computed efficiently, thus, requiring a small amount of additional computation.

To construct a detection model with the generalization ability, we train the detection model with FGSM examples and conduct the detection using other perturbation methods. We adopt the two norm variations in Huang et al. (2017) and define the norm constraint of perturbations as follows:


(3) $$\begin{array}{l} \begin{array}{c} \delta_{2}=\epsilon \sqrt{d}*\frac{\nabla_{s_{t}}J\left(Q_{t},Q_{t-1}+r_{t}\right)}{||\nabla_{s_{t}}J\left(Q_{t},Q_{t-1}+r_{t}\right)|{|}_{2}},\\ \delta_{1}=\text{perturb highest}|\nabla_{s_{t}}J\left(Q_{t},Q_{t-1}+r_{t}\right)|\text{dimension}. \end{array} \end{array}$$

**Attack with smaller frequency**. The strategically-timed attack (Lin et al., 2017) aims to decrease the attack frequency without sacrificing the performance of the un-targeted reinforcement attack. We formally present it below:


(4) $$\begin{array}{l} \begin{array}{c} \delta_{t}=\delta_{t}*c_{t}\;c_{t}\in \left\{0,1\right\},\\ \frac{\sum_{t=1}^{T}c_{t}}{T}<d, \end{array} \end{array}$$

where *c*_*t*_ is a binary variable that controls when to attack; *d* < *T* is the frequency of adversarial examples. There are two approaches to generate the binary sequence *c*_1:*T*_ optimizing a hard integer programming problem and generating sequences *via* heuristic methods. Let *p*_0_, *p*_1_ be the two maximum probability of a policy π, we define *c*_*t*_ as follows, which is different from Lin et al. (2017):


$$c_{t}=\left(p_{0}-p_{1}\right)>threshold.$$

In our experiments, we let the RL-based recommendation system have a peak probability at the maximum action to test the importance of the action to attackers using the above formula. In contrast to the above methods, JSMA and Deepfool are based on the gradient of actions rather than the gradient of *Q* value. One key component of JSMA is saliency map computation used to decide which dimension of vectors (in Image classification is pixels) are modified. Deepfool pinpoints the attack dimension by comparison of affine distances between some class and temporal classes. More details can be found in Papernot et al. (2016) and Moosavi-Dezfooli et al. (2016).

**Counterfactual Based Attack**. Counterfactual can find a similar version of the query input within some distributions, changing the model decisions and receiving a different classification. This helps to explain why a specific classification decision was made by a model and improve the interpretation of model boundaries (Yang et al., 2021), which is known as counterfactual explanations. Recent study reveals that counterfactual explanations produce adversarial examples in deep neural networks (Browne and Swift, 2020). Therefore, we propose to generate counterfactual user interacting processes to be the counterfactual-based attacks for the RL model.

Most of the adversarial examples are generated by adding perturbations. The counterfactual-based attack is recognized as one sub-type of adversarial example, which is different from traditional perturbations. One of the majority differences is that the counterfactual-based attack is generated by causal reasoning. With a casual relationship, we can perform interventions on causes to get counterfactual outcomes. To capture the casual relationships, we introduce Structural Causal Model(SCM) *M* = 〈*U, V, F*〉, given by a directed acyclic graph (DAG) G, where:

- *U* = (*U*_1_, …, *U*_*N*_) is a set of exogenous variables determined by unobserved and omitted factors. We assume that these noises are independent variables such that *U*_*i*_ is independent of all other noise variables.
- *V* = (*V*_1_, …, *V*_*N*_) is a set of endogenous variables that are observed nodes in the DAG.
- *F* = (*f*_1_, …, *f*_*N*_) is a set of structural equations representing the set of edges in the DAG. Each represents a causal mechanism that expresses the value of *V*_*i*_ as a function of the values of parents of *V*_*i*_ in G and the noise term, that is *V*_*i*_ = *f*_*i*_(*Pa*(_*V*_*i*_)*i*_, *U*_*i*_).

To simplify counterfactual reasoning, we assume that the input states follow the Local Causal Models (LCMs) (Pitis et al., 2020), stating that *V*_*j*_ is a parent node of *V*_*i*_ in G if and only if there is a direct edge from *V*_*j*_ to *V*_*i*_ such that setting the value of *V*_*j*_ will have a direct effect on *V*_*i*_ through *f*_*i*_. With this assumption, a large subspace L often exists for each pair of nodes (*Pa*(_*V*_*i*_)*j*_, *V*_*i*_) in the DAG, in which two components are causally independent conditioning on a subset of parents nodes of *V*_*i*_ so that can be considered separately for training and inference.

Specifically, given two states with the same local factorization, we find the similar components of these two states. The similar components remain unchanged in the MDP process, representing that the critical components containing user identifiable information remain. Then, we test whether two sub-states without critical components, *s*_*ir*_ and *s*_*jr*_, are locally independent. By performing interventions on one sub-state without critical components *s*_*jr*_ in SCM, we can calculate the new value of another sub-state *s*_*ir*_. If the difference between the value after intervention and the original value of *s*_*ir*_ is within certain limits, setting the value of *s*_*jr*_ does not have a direct effect on *s*_*ir*_ through the causal mechanism. In that case, we conclude that the two sub-states without critical components are locally independent according to the LCMs. Leaving the critical components untouched, we produce a new counterfactual-based attack by swapping the two locally independent subsets of states *s*_*i*_ and *s*_*j*_. The algorithm is given in Algorithm 1. This process can be interpreted by making intervention $do\left(S_{t}^{i,...,j}\right)=S_{t^{\prime }}^{i^{\prime },...,j^{\prime }}$ on the LCMs $M^{L}$ to obtain the simulation result (Pitis et al., 2020).

**Algorithm 1:** Counterfactual State Generation.

1:	**procedure** Independent(SCM *M*, sub-state *s*_*ir*_, sub-state *s*_*jr*_)
2:	Set the value of *s*_*jr*_ to be $s_{jr}^{\prime }$ ⊳ $s_{jr}^{\prime }$ has random value
3:	Calculate $s_{ir}^{\prime }$ by apply SCM *M* on $s_{jr}^{\prime }$
4:	return true if the $s_{ir}^{\prime }$ has the similar value with *s*_*ir*_ ⊳ *s*_*ir*_ and *s*_*jr*_ are locally independent
5:	**end procedure**
6:	**procedure** Generation(SCM *M*, Current state *s*_*i*_, a random state *s*_*j*_)
7:	Find the similar component *s*_*same*_ in *s*_*i*_ and *s*_*j*_
8:	*s*_*ir*_ ← *s*_*i*_\*s*_*same*_
9:	*s*_*jr*_ ← *s*_*j*_\*s*_*same*_
10:	**if** Independent(*M*, *s*_*ir*_, *s*_*jr*_) is true **then**
11:	Generate counterfactual state $s_{i}^{\prime }$ by swapping locally independent *s*_*ir*_ and *s*_*jr*_
12:	**end if**
13:	**return** counterfactual state $s_{i}^{\prime }$
14:	**end procedure**

### 3.3. Detection Model

The detection model is a supervised classifier, which detects adversarial examples based on the actions of the reinforcement agent in a general feature space. Action-based detection exploits the fact that the defense can be constructed ignoring the attack type. Because state-based detection requires to model the distribution shift of various methods that increases the difficulty of modelling. Suppose the action distributions of an agent are shifted by adversarial examples (Section 4 shows statistical evidence of the drift). Given an abnormal action sequence *a* = π^*^(*a*|*s* + δ) or a counterfactual action sequence, the detection model aims to establish a separating hyperplane between adversarial examples and normal examples, thereby measuring the probability *p*(*y*|*a*, θ) or *p*(*y*|π^*^, *s*, δ, θ), where *y* is a binary variable indicating whether the input data are attacked.

To detect the adversarial examples presented in the last section, we employ an attention-based classifier. We first conduct statistical analysis on the attacked actions whose result is shown in Section 4. The detection model consists of two parts. The first is an encoder to encode the action methods into a low-dimensional feature vector. The second is a classifier to separate different data. We adopt this encoder-decoder model to make a bottleneck and filter out noisy information. The formulation of GRU is as follows:


(5) $$\begin{array}{l} \begin{array}{c} z_{t}=\sigma_{g}\left(W_{z}a_{t}+U_{z}h_{t-1}\right),\\ r_{t}=\sigma_{g}\left(W_{r}a_{t}+U_{r}h_{t-1}\right),\\ \hat{h_{t}}=tanh\left(W_{h}a_{t}+U_{h}◦h_{t-1}\right),\\ h_{t}=\left(1-z_{t}\right)◦h_{t-1}+z_{t}◦\hat{h_{t}}. \end{array} \end{array}$$

We use an action sequence *a*_1:*T*_ to denote a series of user relation vectors or item embedding vectors and apply a recurrent model to encode the temporal relation into the feature vectors. We further adopt a single layer GRU network as our encoder and employ the attention-based dense net for detecting adversarial examples (formulated below).


(6) $$\begin{array}{l} \begin{array}{c} \alpha_{t}=Softmax{\left(W_{e}e+b_{e}\right)}_{t},\\ att,hid=\overset{T}{\underset{t=1}{\sum }}\alpha_{t}h_{t},\\ p=Softmax\left(W_{att}att+b_{att}\right), \end{array} \end{array}$$

where *e* is the combined vector of action embedding and hidden states *hid*—we compute attention weights from embedding vectors and employ a liner unit to distribute probabilities to input time steps; *h*_*t*_ is the output of the encoder. The vectors processed through the attention layer are then piped into a linear unit with softmax to compute the probability of adversarial examples. The loss function is the cross entropy between the true label and corresponding probability,


$$J\left(Att\left(a_{1:T}\right),y\right)=-y◦log\left(p\right).$$

## 4. Experiments

In this section, we report our experiments to evaluate attack methods and our detection model. We first introduce the datasets and then provide quantitative evaluation and discussion on different attacks and our detection model.

### 4.1. Dataset and Experiment Setup

We conduct experiments based on two RL interactive recommendation systems. Following Chen et al. (2019) and Xian et al. (2019) over the real-world dataset – Amazon dataset (He and McAuley, 2016). This public dataset contains user reviews and metadata of the Amazon e-commerce platform from 1996 to 2014. We utilize three subsets named Beauty, Cellphones, and Clothing as our dataset. We directly use the dataset provided by Xian et al. (2019) on Github to reproduce their experiments. Details about Amazon dataset analysis can be found in Xian et al. (2019).

We conduct our attack and detection experiments based on (Xian et al., 2019). We preprocess the dataset by filtering out feature words with higher TF-IDF scores than 0.1. Then, we use 70% data in each dataset as the training set (and the rest as the test set) and the actions of the reinforcement agent as the detection data. We define the actions of PGPR (Xian et al., 2019) as heterogeneous graph paths that start from users and have a length of 4. The three Amazon sub-dataset (Beauty, Cellphones, and Clothing) contain 22,363, 27,879, and 39,387 users. To accelerate experiments, the first 10,000 users of each dataset are extracted for adversarial example production. Users in Beauty get on average 127.51 paths. The counterparts for Cellphones and Clothing are 121.92 and 122.71. We adopt the action file of *l*_∞_ attack with an epsilon of 0.5 as the training set. As the number of paths is large, we utilize the first 1,00,000 paths for train and validation. The ratio of train validation is 80/20. Regarding the test, 1,00,000 paths from each action file are randomly sampled as the test set.

We slightly modify JSMA and Deepfool for our experiments—we create the saliency map by calculating the product of the target label and temporal label to achieve both effectiveness and higher efficiency (by 0.32 s per iteration) of JSMA; We also use sampling to decrease the computation load on a group of gradients for Deepfool. Besides, we set the hidden size of the GRU to 32 for the encoder, the drop rate of the attention-based classifier to 0.5, the maximum length of a user-item path to 4 [according to Xian et al. (2019)], and the learning rate and weight decay of the optimization solver, Adam, to 5e-4 and 0.01, respectively.

### 4.2. Attack Experiments

This section reports our experiments on adversarial attacks. The first part shows the attack experiment results, followed by an analysis of the impact of attack frequency, attack intensity, and the action space of the recommendation system on the attack performance.

**Adversarial attack results**. We are interested in how vulnerable the agent is to perturbation in semantic embedding space. We consider an attack to be effective if a small perturbation leads to a notable performance reduction. We experimentally compare the performance of different attack methods (described in Section 3) in Table 1.

**Table 1:** Adversarial attack results on Amazon Beauty, Cellphone, and Clothing.

**Data**	**Method**	**Parameters**	**NDCG**	**Recall**	**HR**	**Precision**
	Original	-	5.449	8.324	14.401	1.707
Beauty	FGSM *l*_1_	ϵ = 0.1	2.695	3.714	6.599	0.693
	FGSM *l*_2_	ϵ = 1.0	4.567	6.555	13.751	1.653
	FGSM *l*_*inf*_	ϵ = 0.5	2.830	3.909	7.351	0.787
	Counterfactual	-	5.324	8.089	14.077	1.658
	JSMA	-	2.984	3.844	8.254	0.931
	Deepfool	-	3.280	4.352	9.548	1.050
Cellphone	Original	-	3.559	5.510	9.031	0.933
	FGSM *l*_1_	ϵ = 0.1	2.146	3.007	4.832	0.493
	FGSM *l*_2_	ϵ = 1.0	3.000	4.444	7.471	0.784
	FGSM *l*_*inf*_	ϵ = 0.5	2.495	3.312	5.812	0.599
Cloth	Original	-	3.104	4.962	8.292	0.853
	FGSM *l*_1_	ϵ = 0.1	1.765	2.266	3.484	0.350
	FGSM *l*_2_	ϵ = 1.0	3.136	5.019	8.282	0.847
	FGSM *l*_*inf*_	ϵ = 0.5	1.449	2.005	2.865	0.286

We reuse the evaluation metrics of the original model, namely Normalized Discounted Cumulative Gain (NDCG), Recall, Hit Ratio (HR), and Precision for evaluation on the amazon dataset. Table 1 shows the attack results share the same trend with the distribution discrepancy in Table 2. Most attack methods significantly reduce the performance of the reinforcement system. FGSM *l*_1_ achieves the best performance. It reveals that attacks on a single dimension can change the neural network's action drastically. Compared with *l*_1_ and *l*_*inf*_ methods, FGSM *l*_2_ is less effective on three datasets, where the evaluation metrics are mostly the same in contrast to the case without an attack (The original baseline in Table 2). It is worth mentioning that counterfactual attack does not perform well as the others. One of the possible reasons is that the generated counterfactual state still falls in the original latent space. The counterfactual attack can introduce noise to the current state by introducing irrelevant information from future states.

**Table 2:** MMD between a benign distribution and adversarial distribution on Amazon Beauty.

**Data**	**Parameters**	**MMD-org**	**MMD-*l*_1_**
Original	-	0.121	0.620
FGSM *l*_1_	ϵ = 0.1	0.604	0.010
FGSM *l*_2_	ϵ = 1.0	0.016	0.573
FGSM *l*_*inf*_	ϵ = 0.5	0.570	0.011
Counterfactual	-	0.232	0.273
JSMA	-	0.412	0.034
Deepfool	-	0.177	0.458

**Figure 2:**
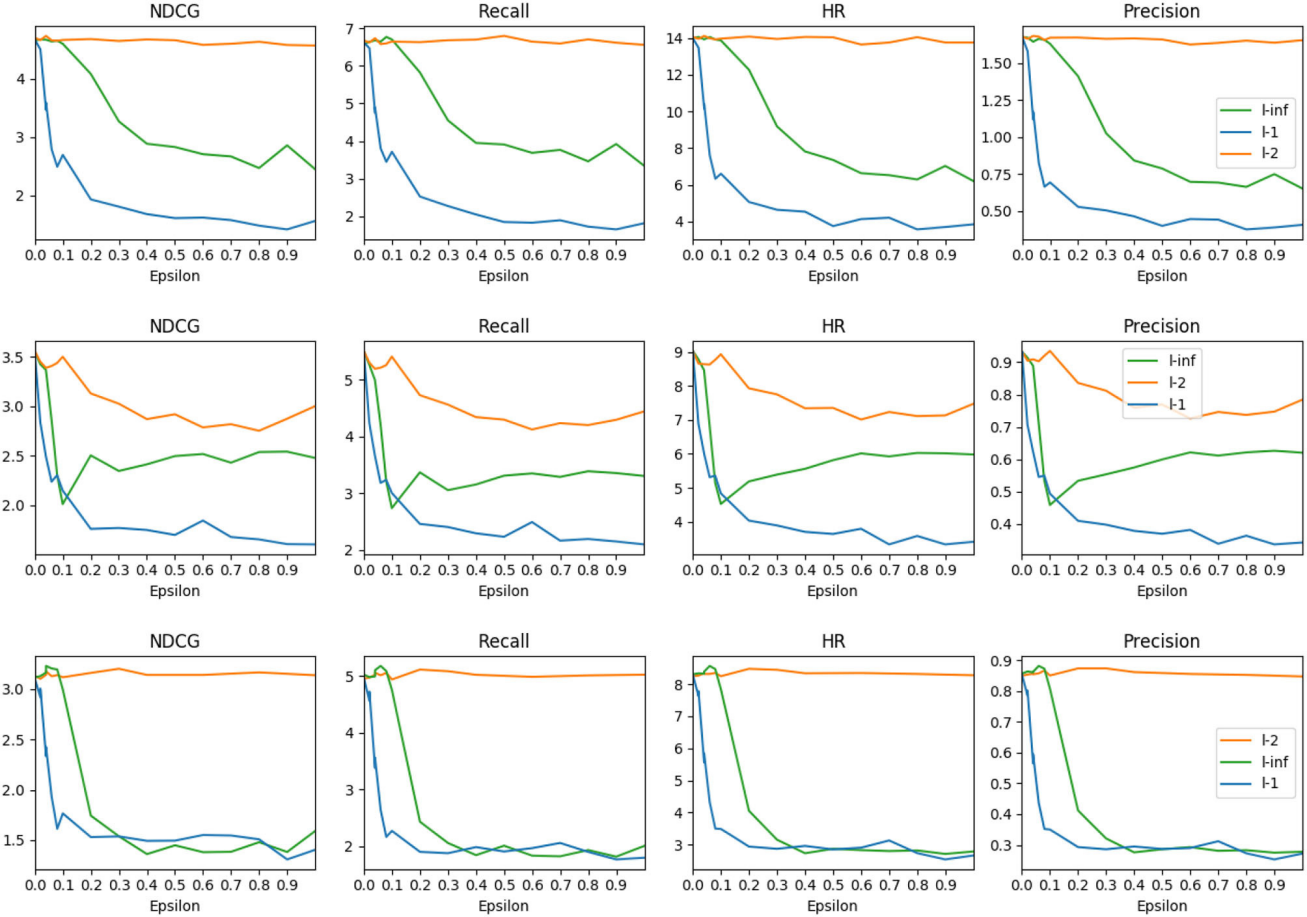
Comparison of three attack methods, *l*_∞_, *l*_2_, and *l*_1_ on three datasets (from top to bottom): Amazon Beauty, Amazon Cellphones, and Amazon Clothing.

**Impact of attack intensity**. Adversarial examples make small perturbations to achieve notable changes in recommendation performance. Although larger perturbations on user-item interaction embeddings are not easily perceptible by humans, decreasing attack intensity might degrade attack effectiveness. To demonstrate the impact of different attack intensities in the context of RL-based recommender systems, we conduct the empirical experiment by varying the attack intensity, which is reflected by the ϵ parameter shown in Equation 2 and Equation 3. Experiment results of attack with epsilon variation of FGSM attack methods on three Amazon datasets (Figure 2) show that compared to a 0.0 value epsilon, all metric values decline as Epsilon increases, and *l*_1_ attack achieves the best result. *l*_1_ follows a similar yet more abrupt trend than the *l*_∞_ attack, while the *l*_2_ attack achieves the worst performance regardless of the epsilon value. Huang et al. (2017) propose to attack the RL applied to games such as Atari. Their experiments reveal that the *l*_2_ attack achieves comparable performance as *l*_1_ and *l*_*inf*_ attacks do. To exclude the possibility that the *l*_2_ might be more effective with larger epsilon values, we set ϵ to 20 to test, but the result is the same. This observation reveals that the attack in user-item-feature embedding space shows different characteristics from attacks in the pixel space.

Another interesting observation is that the metric values show different trends depending on the datasets—unlike on Beauty and Cellphones, the *l*_∞_ attack achieves comparable performance to *l*_1_ on the Clothing dataset when the ϵ is larger than 0.3. The result on the Cellphones dataset shows that the effectiveness of the *l*_*inf*_ attack diminishes as the ϵ continues increasing beyond 0.1.

**Figure 3:**
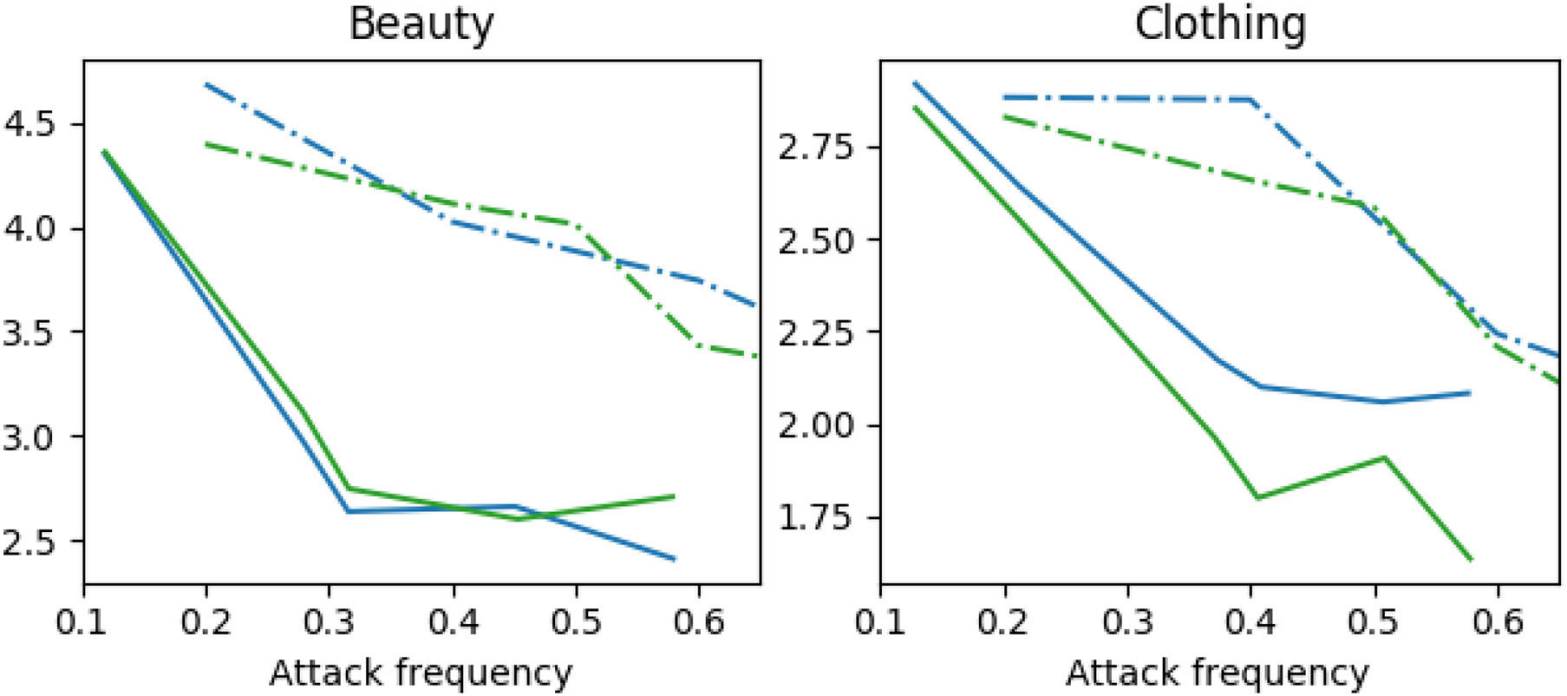
Normalized Discounted Cumulative Gain of attack frequency on Amazon Beauty and Clothing. Dashdot lines represent random attacks, solid lines are strategically-timed attacks. Blue lines are FGSM *l*_*inf*_ attacks, green lines are FGSM *l*_1_ attacks.

**Impact of attack frequency**. We conduct two experiments on attack frequency, random attack, and strategic attack. In the random attack method, the adversarial examples are crafted with a frequency parameter, *p*_*freq*_. In the strategically-timed attack, the adversarial examples are generated by the method shown in Section 3.2. The NDCG metric is presented in Figure 3; other metrics have a similar trend. It can be seen from Figure 3 that the random attack performs worse than the strategically-timed attack. Generating strategically adversarial examples in one-third to half time steps achieves a significant reduction in all metrics.

### 4.3. Detection Experiments

**Analysis of adversarial examples**. We use Maximum Mean Discrepancy as statistical measures of high dimensional data distribution distance. This divergence is defined as:


$$MMD\left(k,X_{org},X_{adv}\right)=\underset{k\in K}{\sup}\left(\frac{1}{n}\overset{n}{\underset{i=1}{\sum }}k\left(x_{org,i}\right)-\frac{1}{m}\overset{n}{\underset{i=1}{\sum }}k\left(x_{adv,i}\right)\right),$$

where *k* is the kernel function, i.e., a radial basis function, which measures the distance between the means of two distributions (Table 2 shows the results); *X*_*org*_, *X*_*adv*_ are benign and adversarial examples. The examples here refer to the actions generated by attacked RL agents. The distribution discrepancy of input data pierces through the deterministic model and shifts the action distribution that would be exploited by the detector. The data is randomly sampled from generated action embeddings of interactive recommendation systems. Each MMD is computed by averaging 40 batches of 500 samples. The actions generated by the RL agent are paths of the users-relation-item graph. As mentioned in Section 4.1, each user gets over 100 paths, which determines the overlapping of original data and adversarial examples decrease with the time step of the path growing. We choose the embedding of the last step to represent the recommended items.

MMD-org shows the discrepancy between the original and adversarial datasets, where MMD-*l*_1_ presents the discrepancy between different attack methods. The results (Table 2) show that the adversarial distribution is different from the original distribution. Also, the disturbed distributions are closed to each other regardless of the attack type. This insight clarifies that we can use a classifier to separate benign data and adversarial data, and it can detect several attacks simultaneously, which might be transferred to other reinforcement learning attack detection tasks.

**Detection Performance**. From a statistical perspective, the above analysis shows that one classifier can detect multiple types of attacks. We evaluate the detection performance of different models using Precision, Recall, and F1 score.

We adopt an attention-based network for detection experiments. The detection model is trained on the FGSM *l*_1_ attack with ϵ at 0.1 for all datasets. The results (Table 3) show that our detection model achieves better performance on attacks that cause serious disruption. The detection precision and recall rise as the attack is stronger. *l*_∞_ attack validates this trend, which shows that our model can detect weaker attacks as well. The result of detection on *l*_2_ attack can be reasoned with the MMD analysis shown above. High precision and low recall show that most *l*_2_ adversarial examples are close to benign data, which confuses the detector. The *l*_1_ attack with ϵ = 1.0 validates our detector performs well yet achieves worse performance on other tests on the Cellphones dataset. Our model can also detect the counterfactual-based attack since the data distribution has been changed, verifying that our detection model can detect different types of attacks. Our results on factor analysis (Table 3) show that the detection model can detect attacks even under low attack frequencies. But the detection accuracy decreases as the attack frequency drops—the recall decreases significantly to 40.1% when 11.8% of examples represent attacks.

**Table 3:** Detection result and factor analysis.

**Dataset**	**Attack**	**Precision**	**Recall**	**F1 Score**
Beauty	*l*_1_ 0.1	0.919	0.890	0.904
	*l*_2_ 1.0	0.605	0.119	0.199
	*l*_*inf*_ 0.5	0.918	0.871	0.894
	Counterfactual	0.900	0.895	0.898
	JSMA	0.910	0.793	0.848
	Deepfool	0.915	0.840	0.876
Cellphones	*l*_1_ 0.1	0.801	0.781	0.791
	*l*_2_ 1.0	0.754	0.593	0.664
	*l*_*inf*_ 0.5	0.795	0.752	0.773
	*l*_1_ 1.0	0.810	0.825	0.817
Clothing	*l*_1_ 0.1	0.911	0.866	0.888
	*l*_2_ 1.0	0.541	0.099	0.168
	*l*_*inf*_ 0.5	0.912	0.879	0.895
Dataset	Frequency	Precision	Recall	F1 Score
Beauty	*l*_1_ 0.02	0.823	0.362	0.503
	*l*_1_ 0.04	0.906	0.754	0.823
	*l*_1_ 0.06	0.915	0.841	0.876
	*l*_1_ 0.08	0.918	0.872	0.894
	*l*_1_ 0.3	0.922	0.927	0.924
Dataset	Frequency	Precision	Recall	F1 Score
Beauty	*l*_1_ 0.579	0.921	0.912	0.917
	*l*_1_ 0.451	0.921	0.908	0.914
	*l*_1_ 0.316	0.918	0.879	0.898
	*l*_1_ 0.279	0.914	0.829	0.869
	*l*_1_ 0.118	0.837	0.401	0.543

## 5. Conclusion

Adversarial attacks on reinforcement agents can greatly degrade user experience in interactive recommendation systems, as an intervention on causal factors can result in a different recommended result. In this article, we systematically study adversarial attacks on RL-based recommendation systems by investigating different attack methods and the impact of attack intensity and frequency on the performance of adversarial examples. We conduct statistical analysis to show that classifiers, especially an attention-based detector, can well separate the detection data. Our extensive experiments show the excellent performance of our attack and detection models.

## Data Availability Statement

The original contributions presented in the study are included in the article/Supplementary Materials, further inquiries can be directed to the corresponding author.

## Author Contributions

SW and YC write the original draft, conception, and design the experiments. XC provides the inspection and validation of the experiments. LY provides the supervision and manuscript reviewing. XW and QS provide manuscript reviewing. All authors contributed to the article and approved the submitted version.

## Conflict of Interest

The authors declare that the research was conducted in the absence of any commercial or financial relationships that could be construed as a potential conflict of interest.

## Publisher's Note

All claims expressed in this article are solely those of the authors and do not necessarily represent those of their affiliated organizations, or those of the publisher, the editors and the reviewers. Any product that may be evaluated in this article, or claim that may be made by its manufacturer, is not guaranteed or endorsed by the publisher.
