# Supplementary figures and images for: Adversarial Robustness of Deep Reinforcement Learning Based Dynamic Recommender Systems
Source: Front Big Data. 2022 May 3;5:822783. doi: 10.3389/fdata.2022.822783 (PMC9110778; doi:10.3389/fdata.2022.822783)

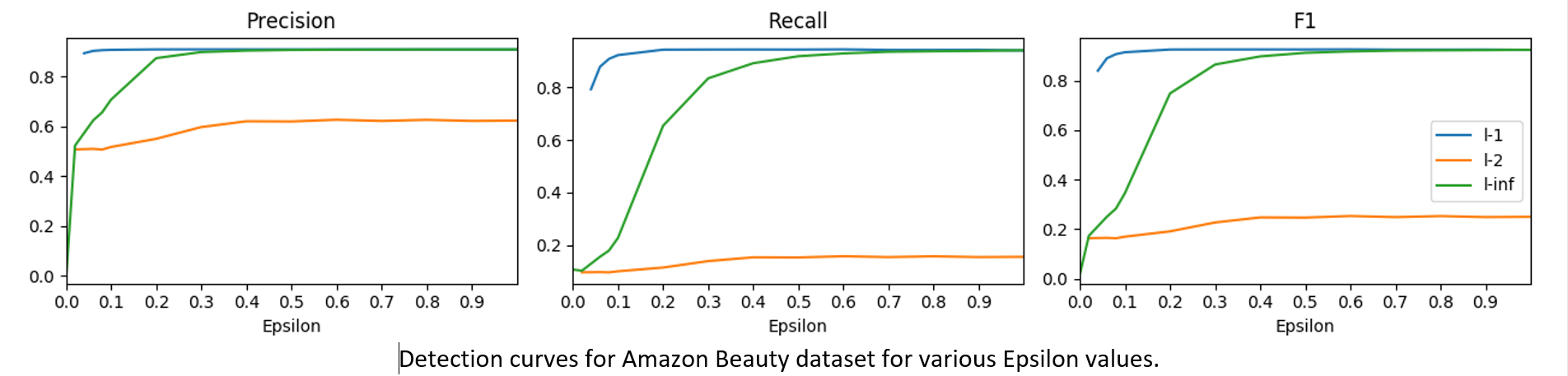

Supplement: Supplementary file 1 [file Image_1.png]

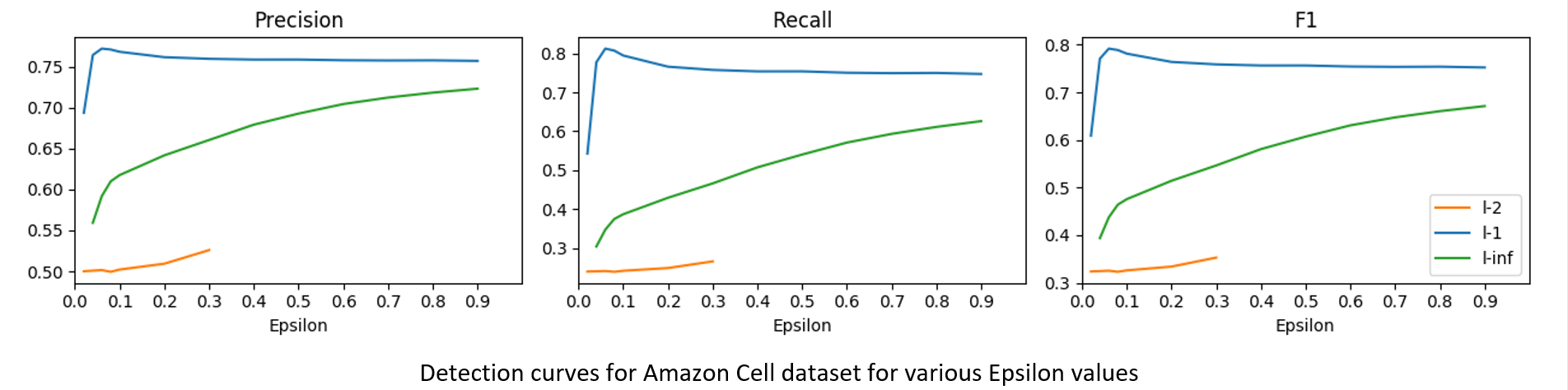

Supplement: Supplementary file 2 [file Image_2.png]

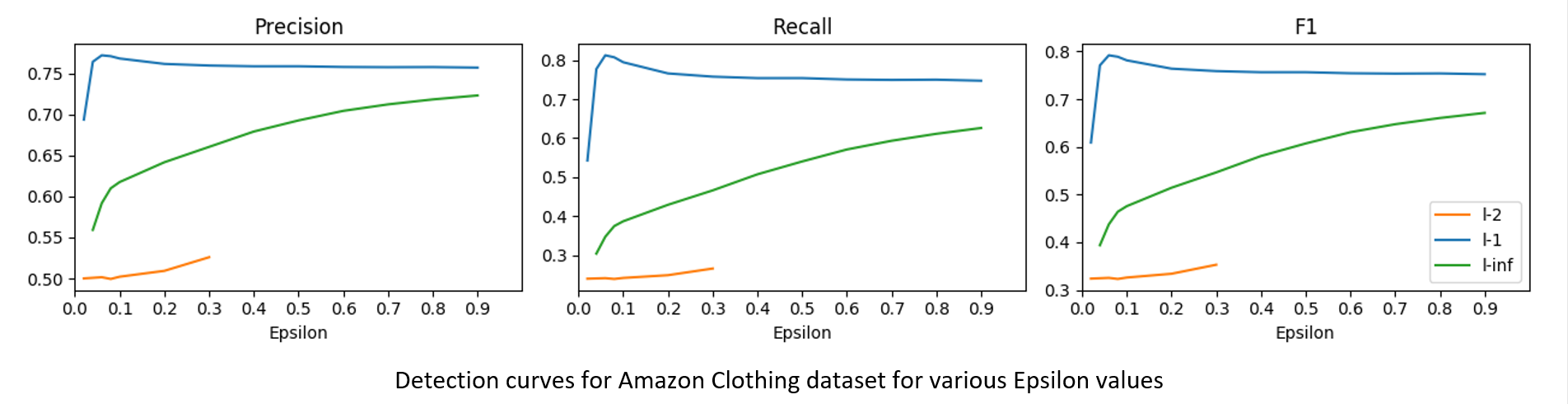

Supplement: Supplementary file 3 [file Image_3.png]
